# Hepatitis B virus core immunoglobulin M: false positive more common than true

**DOI:** 10.1128/jcm.00525-26

**Published:** 2026-06-12

**Authors:** Marie L. Landry, Paul E. Bernard

**Affiliations:** 1 Department of Laboratory Medicine, Yale University School of Medicine 5755, New Haven, Connecticut, USA; 2 Department of Medicine, Yale University School of Medicine 5755, New Haven, Connecticut, USA; St Jude Children's Research Hospital, Memphis, Tennessee, USA

**Keywords:** hepatitis B virus, anti-HBc IgM, HBV window, HBV diagnosis

## LETTER

Hepatitis B virus (HBV) infection is most commonly diagnosed by testing patients’ blood for HBV antigens and antibodies. Hepatitis B surface antigen (HBsAg) and antibody (anti-HBs) were the first tests developed. However, it was soon noted in acute HBV infections that HBsAg became negative several weeks before anti-HBs was detected, yet blood remained infectious in the interim (1–3). This “seronegative window” contributed to inadvertent administration of HBV-infected blood. To fill in this diagnostic gap, assays to detect total and IgM antibodies to hepatitis B core (HBc) antigen were developed (4, 5). Subsequently, anti-HBc IgM has been routinely employed to diagnose acute HBV infection.

In the Clinical Virology Laboratory at Yale New Haven Hospital, HBV serology is performed on the widely used Abbott ARCHITECT platform (Abbott Laboratories, Abbott Park, IL, USA). Over 5,000 samples are tested for anti-HBc IgM, and ~0.5% are positive each year. Since IgM assays are prone to false positivity (6), we investigated the accuracy of anti-HBc IgM.

**Fig 1:**
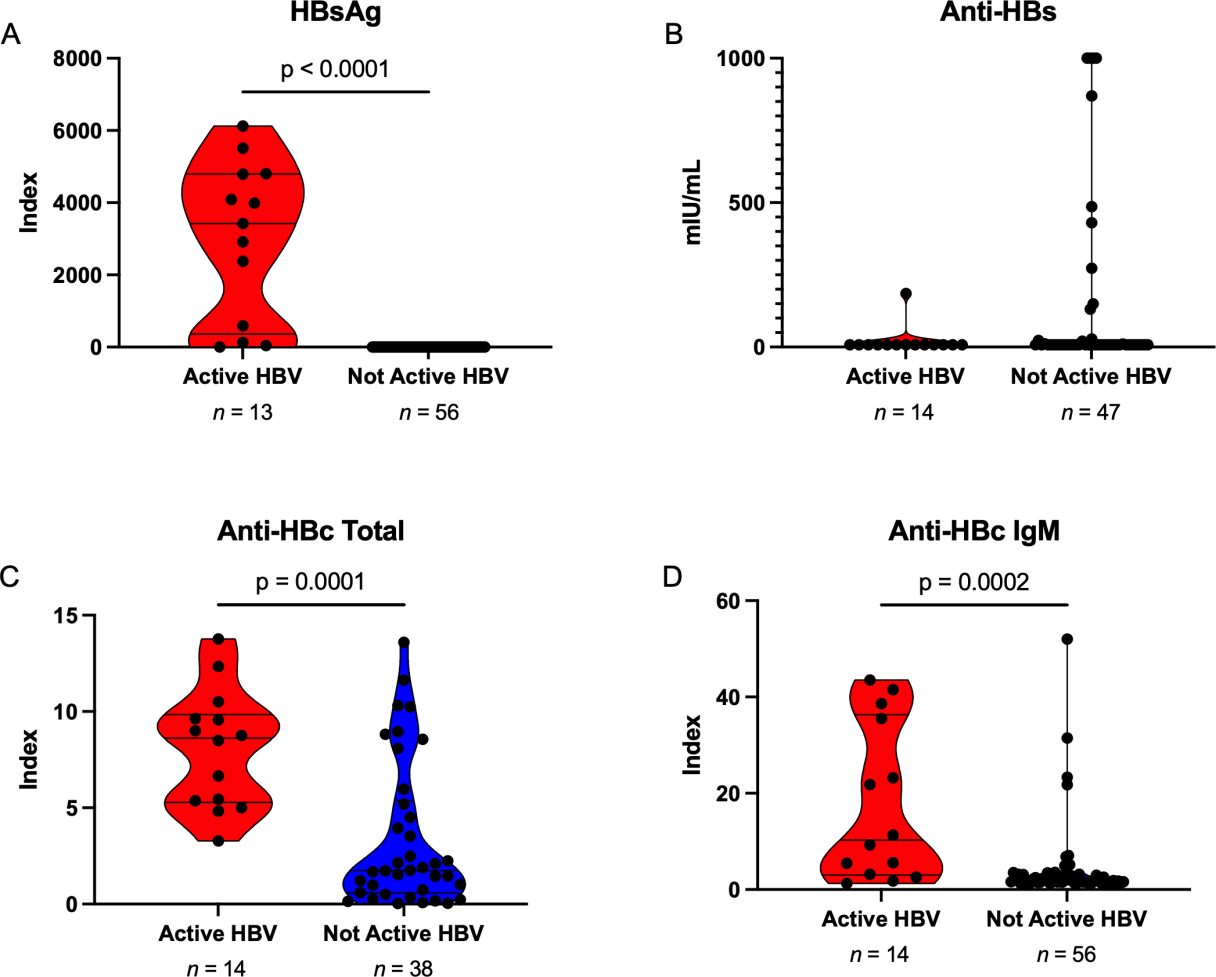
Hepatitis B virus serology: comparative reactivity for anti-HBc IgM-positive patients with and without active HBV infection. (**A**) Hepatitis B surface antigen (HBsAg); (**B**) hepatitis B surface antibody (anti-HBs); (**C**) hepatitis B core total antibody (anti-HBc total); and (**D**) hepatitis B core IgM antibody (anti-HBc IgM). Note: Not all tests were performed in all patients; the number of patients tested is included in each panel. For HBsAg (**A**), one result in an active HBV patient was reported as positive by an outside laboratory without an index value and thus could not be included in the graph (*n* = 13). For anti-HBs (**B**), results of <8.00 are given as zero and results of >1,000 are given as 1,000. Statistics using the Mann-Whitney test.

Charts were reviewed on all 70 unique patients with samples positive for anti-HBc IgM from January 2023 to March 2026. Results of initial serology tests are shown in Table 1. Serial tests were excluded. Reactivity for the four HBV serology tests is shown in Fig. 1A through D.

**TABLE 1:** Hepatitis B serology and HBV DNA results for 70 anti-HBc IgM-positive patients

Active HBV infection	No. with results	HBsAg	Anti-HBs	Anti-HBc total	Anti-HBc IgM	HBV DNA PCR No. of Pos/No. of tested
	13	+	−	+	+	12/12
	1	+	+	+	+	1/1
Total	14					13/13

Not active HBV infection^*a*^						
	19^*b*^	−	−	+	+	0/15
	6	−	−	−	+	0/5
	5	−	+	−	+	0/4
	6	−	+	+	+	0/5
	4	−	+	Not done	+	0/3
	7	−	−	Not done	+	0/1
	7	−	Not done	Not done	+	0/3
	2	−	Not done	+	+	0/0
Total	56					0/36

^*a*^ HBV infection not subsequently suspected or confirmed.

^*b*^ HBV “window” pattern of reactivity with no evidence for HBV infection.

Only 14 patients (20%) were found to have active HBV infection. All 14 had positive HBsAg; 13 out of 14 were also tested by HBV DNA PCR, and all were positive. Seven had known HBV infection, and seven were new diagnoses. Two newly infected patients had serial samples tested, and the HBsAg and anti-HBs seronegative window was not observed. Rather, with the ARCHITECT assay, HBsAg and anti-HBs positivity overlapped. For active HBV-infected patients, 10 out of 14 samples had anti-HBc IgM indices >5.00 (median 10.30); lower indices were associated with chronic HBV infections (Fig. 1D).

The other 56 anti-HBc IgM-positive results (80%) were likely false positives in patients without active HBV (Table 1). HBV DNA was not detected in the 36 patients in this group tested by PCR. For these patients without active HBV infection, anti-HBc IgM reactivity was typically low; 50 out of 56 samples (89.3%) had an anti-HBc IgM index <5.00 (median 1.94). Higher IgM reactivity was associated with Waldenström’s macroglobulinemia, monoclonal gammopathy of uncertain significance (MGUS), primary EBV, and primary CMV (Fig. 1D). Clinical features for patients with false-positive anti-HBc IgM results are summarized in Table 2.

**TABLE 2:** Clinical features of patients without active HBV infection and with false-positive anti-HBc IgM results^*a*^^,^^*b*^^,^^*c*^

Clinical feature	No.	Examples
Infection, not HBV	30	Chronic HCV, HIV, bacteremia, pneumonia, sepsis, UTI, cellulitis; acute babesia, primary EBV, primary CMV
Malignancy	18	Various malignancies, chemotherapy, Waldenström, MGUS
Transplant recipient	5	Kidney, liver, stem cell transplant
Substance abuse	13	Drugs, alcohol, alcoholic hepatitis
Other liver disease	10	Steatohepatitis, autoimmune hepatitis, cirrhosis, hepatic cyst, hepatomegaly
Other miscellaneous	10	Incidental elevated liver enzymes, fertility treatment, psoriasis biologic treatment, preoperative screen, anemia

^*a*^ Many patients had more than one characteristic.

^*b*^ HBV infection was not subsequently suspected or established in any of these patients.

^*c*^ CMV, cytomegalovirus; EBV, Epstein–Barr virus; HCV, hepatitis C virus; HIV, human immunodeficiency virus; MGUS, monoclonal gammopathy of uncertain significance; UTI, urinary tract infection.

For patients with complete serology, 19 results fit the “window” pattern of HBV with detection of anti-HBc total and IgM antibodies only. More detailed clinical characteristics are provided for these 19 patients in Table 3. For 15 of these patients, HBV DNA PCR was performed and was not detected. For 11 patients, liver enzymes were within normal limits.

**TABLE 3:** Characteristics of 19 patients with “window” pattern of hepatitis B serology^*a*^

Patient	Age/sex	Hepatitis B serology				HBV DNA PCR	Bilirubin and liver enzymes				Clinical characteristics
		HBsAg (≥1.0)	Anti-HBs (≥12.0) mIU/mL	Anti-HBc total (≥0.80)	Anti-HBc IgM (≥1.21)		Total bilirubin (≤1.2 mg/dL)	Alk phos (9–122 U/L)	ALT (9–59 U/L)	AST (10–35 U/L)	
1	63 M	0.14	<8.00	5.87	1.56	Negative	0.5	131	87	69	HCV, prostate cancer, renal transplant, sepsis, and MRSA
2	71 M	0.23	<8.00	2.15	1.39	Negative	0.2	209	96	74	Severe COPD and pneumonia
3	69 M	0.28	<8.00	1.75	1.34	Negative	0.3	148	7	23	Non-Hodgkin lymphoma and incidental HCV
4	59 F	0.31	<8.00	1.02	2.36	Negative	0.4	218	66	45	Abdominal lymphadenopathy and positive ANA
5	90 M	0.57	<8.00	3.94	4.62	Negative	0.5	393	84	52	Septic shock and *Klebsiella* UTI
6	72 M	0.25	<8.00	8.55	1.5	Negative	1.5	82	447	683	HCV, cirrhosis, and varices
7	62 F	0.25	<8.00	1.89	1.25	Negative	7.6	155	40	121	MASH, alcohol use disorder, cirrhosis, and ascites
8	63 F	0.2	<8.00	3.53	1.78	Negative	10.3	227	406	1085	Autoimmune hepatitis, alcohol use disorder, and rhabdomyolysis
9	76 M	0.13	<8.00	2.1	1.64	Negative	Within normal limits				Glioblastoma and cellulitis
10	81 F	0.21	<8.00	2.24	1.63	Negative	Within normal limits				Metastatic lung carcinoma
11	33 M	0.33	<8.00	13.59	2.45	Negative	Within normal limits				Depression with suicide attempt
12	68 F	0.43	<8.00	5.19	1.68	Negative	Within normal limits				HCV, alcohol use disorder, cirrhosis, and septic shock
13	23 M	0.18	<8.00	1.22	7.1	Negative	Within normal limits				Renal transplant recipient
14	66 M	0.29	<8.00	1.68	3.49	Negative	Within normal limits				Chronic HCV
15	68 M	0.29	<8.00	1.53	1.32	Negative	Within normal limits				Atypical CLL and HCV
16	70 F	0.28	<8.00	1.73	2.31	Not done	Within normal limits				Colon cancer on chemotherapy
17	70 F	0.22	<8.00	4.51	3.16	Not done	Within normal limits				HCV and UTI
18	46 F	0.2	<8.00	11.83	1.55	Not done	Within normal limits				HCV, cirrhosis, and varices
19	75 M	0.15	<8.00	1.46	23.32	Not done	Within normal limits				Waldenström macroglobulinemia

^*a*^ Alk phos, alkaline phosphatase; ALT, alanine aminotransferase; ANA, antinuclear antibody; AST, aspartate aminotransferase; CLL, chronic lymphocytic leukemia; COPD, chronic obstructive pulmonary disease; HCV, hepatitis C virus; MASH, metabolic dysfunction-associated steatohepatitis; MRSA, methicillin-resistant *Staphylococcus aureus*; UTI, urinary tract infection.

Anti-HBc IgM-positive results are uncommon but significant. Reference laboratories’ interpretive comments state positive results indicate recent HBV infection, and one adds anti-HBc IgM “is a reliable marker for acute disease. In some cases, hepatitis B core IgM antibody may be the only specific marker for the diagnosis of acute infection with hepatitis B virus.”

However, of 70 anti-HBc IgM-positive patients over a 3-year period in our laboratory, a remarkable 56 (80%) appeared to be falsely positive, including those with the HBV “window” pattern. True anti-HBc IgM-positive patients had HBsAg and HBV DNA detected, whereas false-positive patients did not. Thus, anti-HBc IgM results are best confirmed with antigen or nucleic acid assays to detect the virus. As a result of these findings, our anti-HBc IgM interpretive comments now include the possibility of a false-positive result and the need for confirmation. Although based on a single laboratory and platform, our results support reassessing the accuracy and interpretation of positive anti-HBc IgM results, including the “window” pattern, and confirming HBsAg-negative results by HBV DNA PCR.
